# Aggressive angiomyxoma of the spermatic cord: A rare entity

**DOI:** 10.4103/0970-1591.45555

**Published:** 2009

**Authors:** Ajay Malik, K. J. Singh, Anurag Mehta

**Affiliations:** Department of Pathology, Armed Forces Medical College, Pune-411 040, India; 1 Department of Surgery, Armed Forces Medical College, Pune-411 040, India

**Keywords:** Angiomyxoma, immunohistochemistry and spermatic cord

## Abstract

Aggressive angiomyxoma (AA) is an uncommon tumor occurring in females and is rarely reported in males with propensity to recur. Due to its presence in perineal and genital region, it has to be differentiated from other myxoid neoplasms. The tumor expresses estrogen and progesterone receptors, which may have a role to play in tumor therapy. Wide local excision remains the mainstay of the treatment. We present a case of AA excised from left spermatic cord.

## INTRODUCTION

Aggressive angiomyxoma (AA) is a locally aggressive, distinctly fibromyxoid, slow growing tumor in the genital, perineal and pelvic regions of adult females. It was first reported by Steeper and Rosai.[1] It has been seen in spermatic cord and pelvic region of men.[2] The tumor looks bland on microscopy. It has a propensity for local recurrence and expresses hormonal receptors.[3]

## CASE REPORT

An 18-year-old male presented with a painless swelling of the left hemiscrotum of 6 months duration. No history of trauma, fever, weight loss, abdominal lump or previous surgery was elicited. Clinically, a 10×08 cm sized fluctuant, transilluminant, and nontender, irreducible swelling conforming to a primary left vaginal hydrocele was seen. He was taken up for hydrocelectomy in January 2008. Preoperatively, testis appeared normal in external appearance and consistency. Adherent to the tunica vaginalis was a 6x4 cm ill-defined gelatinous mass extending up towards the spermatic cord. This was excised and sent for histopathology. Postoperative recovery was uneventful and patient is under regular follow-up.

The mass was gray white and gelatinous in appearance. After formalin fixation and embedding in paraffin, 5 µm sections were cut and stained with H and E staining. Additional sections were taken on poly L-lysine coated slides for immunohistochemistry (IHC). The staining intensity was assessed as weak +, moderate ++, and strong +++. MIB 1 and p53 expression counts were performed as percentage positivity.

Histopathology revealed an infiltrative neoplasm composed of widely scattered spindle shaped cells with eosinophilic cytoplasm in an abundant myxoid stroma with numerous variable sized vessels. Focal perivascular collection of neoplastic cells was seen. The tumor did not reveal obvious atypia. Numerous mast cells and occasional cluster of lymphocytes was seen.

The tumor cells demonstrated immunoreactivity for vimentin, CD 34 and focally to PR, desmin and smooth muscle actin, but were negative for CK, S-100, p53, and ER. The tumor revealed a very low MIB 1 expression (< 1%).

## DISCUSSION

AA can be seen in paratesticular soft tissue rarely. It has a propensity for local recurrence(1–4). 43 cases have been described in the males so far with left spermatic cord involvement in 7 cases.[3] The tumor needs to be distinguished from angiomyofibroblastoma (AMFB), myxoid malignant fibrous histiocytoma and myxoid neurofibroma. The distinctive features are shown in Table 1.

**Table 1:** Differential diagnosis

	AA(1–3,7)	AMFB(5,8)	MN(4)	MM
Common site	Perineum	Genitals	Extremity	Extremity
Recurrence	Yes	No	No	Yes, With metastasis
Size (cm)	<20	V	V	<10
Circumscription	Absent	Present	Present	Absent
Cellularity	Hypocellular	V	Hypocellular	V
Vessels	Variable size	Thin	Small	Curvilinear and thin
Cell type	Spindle	Spindle	Spindle	Fusiform, stellate
Mitosis	Infrequent	Absent	Absent	Present
Inflammation	Mast cells	Absent	Absent	Mixed
Perivascular whorls	Present	Present	Absent	Absent
Immunohistochemistry	Vimentin, desmin, smooth muscle actin, CD34 +, ER/PR+/-(7), S100 -	Vimentin, desmin	S-100 +	Vimentin, CD34 + actin+/-
Behaviour	Recurrence	Benign	Benign	Depends on grade

Present, - absent AA - aggressive angiomyxoma, AMFB - Angiomyofibroblastoma, MN - Myxoid neurofibroma, MM - Myxoid MFH, V - Variable

**Figure 1:**
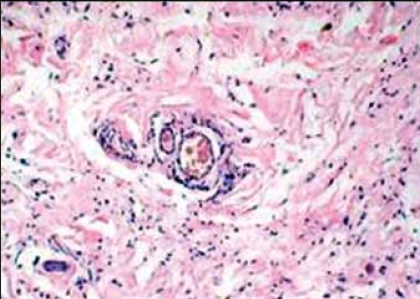
Scanner power view reveals spindle shaped tumor cells in a myxoid matrix (H&E, x10 times magnification, ×10)

**Figure 2:**
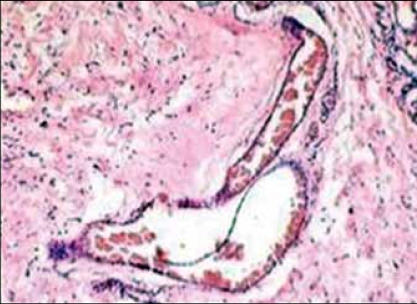
Low power view reveals variable sized vessels (H&E, ×20 times magnification, x20)

**Figure 3:**
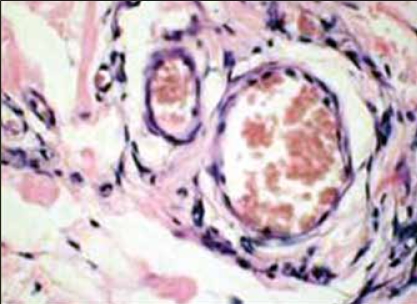
High power view shows perivascular arrangement of spindle shaped cells (H&E, x40 times magnification, ×40)

The histology of the tumor is similar to that seen in female counterparts. The tumor cells are spindle shaped seen scattered in loose myxoid stroma [Figures 1 and 2]. Many vessels of varying caliber are seen surrounded by tumor cells [Figure 3]. Numerous mast cells [Figure 2] were present through out the tumor stroma along with focal lymphoid infiltrate as described also by Garner et al.[8] Steeper and Rosai postulated a myofibroblastic origin of the tumor cells,[1] but recently Martinez et al. in 2003 found a smooth muscle cell origin using electron microscopy.[6]

**Figure 4:**
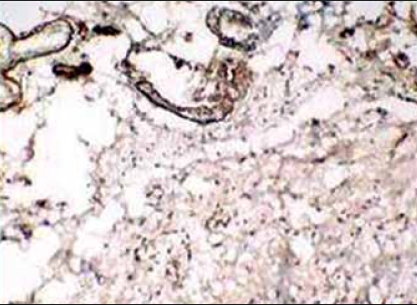
IHC - diffuse vimentin reactivity of tumor cells

**Figure 5:**
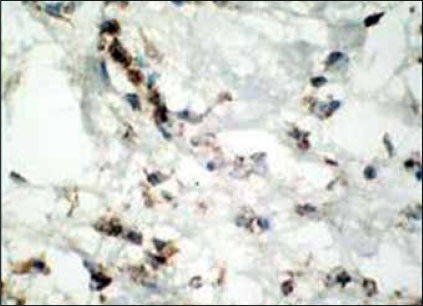
IHC - CD34 positive tumor cells

**Figure 6:**
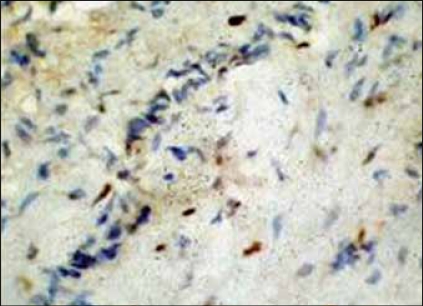
IHC - Scattered progesterone positive tumor cells

The tumor showed focal positivity for desmin and smooth muscle actin. CD34 and vimentin expression was present diffusely throughout the tumor [Figures 4 and 5]. Van Roggen et al. have shown presence of ER,[7] however, our tumor repeatedly did not show any positivity for ER but was positive for PR [Figure 6]. Presence of these receptors denotes a role for antiestrogenic therapy in tumors. A low proliferative index (MIB-1 < 1%) correlates well with the tumor's slow rate of growth. Negativity for S-100 ruled out a neurogenic origin.

Our case is under regular follow up in the surgery outdoor patient department.

To conclude, AA is a locally aggressive tumor seen rarely to involve spermatic cord and expresses hormonal receptors. The mainstay of the treatment is wide local excision.

## References

[1] Steeper TA, Rosai J (1983). Aggressive angiomyxoma of the female pelvis and perineum. Report of nine cases of a distinctive type of gynecologic soft-tissue neoplasm. Am J Surg Pathol.

[2] Tsang WY, Chan JK, Fisher C, Fletcher CD (1992). Aggressive angiomyxoma: a report of four cases occurring in men. Am J Surg Pathol.

[3] Idrees MT, Hoch BL, Wang BY, Unger PD (2006). Aggressive angiomyxoma of male genital region. Report of 4 cases with immunohistochemical evaluation including hormone receptor status. Ann Diagn Pathol.

[4] Holloway P, Kay E, Leader M (2005). Myxoid tumors: a guide to the morphological and immunohistochemical assessment of soft tissue myxoid lesions encountered in general surgical practice. Current Diagn Pathol.

[5] Fletcher CD, Tsang WY, Fisher C, Lee KC, Chan JK (1992). Angiomyofibroblastoma of the vulva: a benign neoplasm distinct from aggressive angiomyxoma. Am J Surg Pathol.

[6] Martinez MA, Ballestin C, Carabias E, González Lois C (2003). Aggressive angiomyxoma: an ultrastructural study of four cases. Ultrastruct Pathol.

[7] van Roggen JF, van Unnik JA, Briaire-de Bruijn IH, Hogendoorn PC (2005). Aggressive angiomyxoma: a clinicopathological and immunohistochemical study of 11 cases with long term follow up. Virchows Arch.

[8] Garnter SR, Nucci MR, Fletcher CD (1997). Aggressive angiomyxoma: reappraisal of its relationship with angiomyofibroblastoma in a series of 16 cases. Histopathology.

